# Comparison of the complications between minimally invasive surgery and open surgical treatments for early-stage cervical cancer: A systematic review and meta-analysis

**DOI:** 10.1371/journal.pone.0253143

**Published:** 2021-07-01

**Authors:** Yilin Li, Qingduo Kong, Hongyi Wei, Yongjun Wang

**Affiliations:** 1 Clinical Medical College, Weifang Medical University, Weicheng District, Weifang, Shandong, China; 2 Division of Gynecology and Obstetrics, Life Science Park of Zhongguancun, Peking University International Hospital, Changping District, Beijing, China; University of Insubria, ITALY

## Abstract

**Background:**

This meta-analysis comprehensively compared intraoperative and postoperative complications between minimally invasive surgery (MIS) and laparotomy in the management of cervical cancer. Even though the advantages of laparotomy over MIS in disease-free survival and overall survival for management of gynecological diseases have been cited in the literature, there is a lack of substantial evidence of the advantage of one surgical modality over another, and it is uncertain whether MIS is justifiable in terms of safety and efficacy.

**Methods:**

In this meta-analysis, the studies were abstracted that the outcomes of complications to compare MIS (laparoscopic or robot-assisted) and open radical hysterectomy in patients with early-stage (International Federation of Gynecology and Obstetrics classification stage IA1-IIB) cervical cancer. The primary outcomes were intraoperative overall complications, as well as postoperative aggregate complications. Secondary outcomes included the individual complications. Two investigators independently performed the screening and data extraction. All articles that met the eligibility criteria were included in this meta-analysis.

**Results:**

The meta-analysis finally included 39 non-randomized studies and 1 randomized controlled trial (8 studies were conducted on robotic radical hysterectomy (RRH) vs open radical hysterectomy (ORH), 27 studies were conducted on laparoscopic radical hysterectomy (LRH) vs ORH, and 5 studies were conducted on all three approaches). Pooled analyses showed that MIS was associated with higher risk of intraoperative overall complications (OR = 1.41, 95% CI = 1.07–1.86, P<0.05) in comparison with ORH. However, compared to ORH, MIS was associated with significantly lower risk of postoperative aggregate complications (OR = 0.40, 95% CI = 0.34–0.48, P = 0.0143). In terms of individual complications, MIS appeared to have a positive effect in decreasing the complications of transfusion, wound infection, pelvic infection and abscess, lymphedema, intestinal obstruction, pulmonary embolism, deep vein thrombosis, and urinary tract infection. Furthermore, MIS had a negative effect in increasing the complications of cystotomy, bowel injury, subcutaneous emphysema, and fistula.

**Conclusions:**

Our meta-analysis demonstrates that MIS is superior to laparotomy, with fewer postoperative overall complications (wound infection, pelvic infection and abscess, lymphedema, intestinal obstruction, pulmonary embolism, and urinary tract infection). However, MIS is associated with a higher risk of intraoperative aggregate complications (cystotomy, bowel injury, and subcutaneous emphysema) and postoperative fistula complications.

## 1. Introduction

Being the fourth most common cancer among women, it has been estimated that there were approximately 528, 000 new cases of cervical cancer with 266, 000 deaths annually [1]. Until now, radical hysterectomy with an open abdominal approach was the predominant modality for the treatment of early cervical cancer [2]. After 1992, with the development of laparoscopic approach, minimally invasive surgery (MIS, i.e., laparoscopy or robotic surgery) for radical hysterectomy to treat cervical cancer has been accepted widely as a standard treatment for early-stage cervical cancer [3].

Surprisingly, the results of Laparoscopic Approach of the Cervix (LACC) clinical trial showed that minimally invasive radical hysterectomy was associated with lower rates of disease-free survival and overall survival compared with open surgery in 2018 [4]. After that, the open abdominal approach was defined as the “standard and recommended approach to radical hysterectomy” for cervical cancer by the National Comprehensive Cancer Network (NCCN) guidelines [5]. Therefore, discussing the surgical complications have to be done clarifying better the actual role of MIS and laparotomy in cervical cancer.

Till date, the advantages of MIS over laparotomy for management of gynecological diseases have been cited in the literature to included less blood loss, shorter hospital stay, and faster recovery [6–8]. Similarly, most previous studies on this subject also showed that robotic surgery has the advantages of providing a three-dimensional perspective and more accurate surgical positioning than laparotomy [9–11]. However, MIS was also associated with its complexity of operation, longer learning curve, and higher cost than laparotomy. Therefore, there is no good evidence of the overall advantage of one surgical modality over another, and it is uncertain whether MIS is justifiable in terms of safety and efficacy, due to the small sample sizes, the low-quality of previous studies, and the limited number of randomized controlled trials (RCTs).

As for complications, many previous studies showed that MIS and open radical hysterectomy (ORH) have no difference in terms of intraoperative and postoperative complications [12]. With further development of instruments and skills, several studies found that MIS was associated with lower rate of intraoperative and postoperative complications than laparotomy [13]. Unfortunately, till date, it is unclear whether the rates of individual complications in MIS are also less than what are seen in laparotomy. Further emphasizing the severity of complications, which are a key factor in the evaluation of cervical cancer.

The aim of this meta-analysis was to compare the published rates of common intraoperative and postoperative complications between ORH and MIS in order to provide valid evidence for evaluating the advantages of different surgical procedures for managing cervical cancer.

## 2. Methods

### 2.1. Search strategy

A comprehensive, systemic search for articles was performed using the databases of PubMed, Embase, Cochrane library, and Web of science. We searched the articles in each database from the data of its inception until—February 2020. Search terms included a combination of synonyms and abbreviations relating to cervical cancer, laparoscopy, laparotomy, robotic surgery, and complication. All articles that met the eligibility criteria were assessed. The details of the search strategy are shown in S1 Table.

### 2.2. Selection criteria

Studies were included if they met the following criteria: (1) Patients were classified as stage IA-IIB (according to the 2018 International Federation of Gynecology and Obstetrics classification); (2) Subjects were females who underwent LRH, laparoscopic-assisted vaginal radical hysterectomy (LAVRH), RAH or ORH as primary treatment for cervical cancer; (3) The outcomes of complications in MIS and ORH were reported. Articles were excluded if they met the following criteria: (1) Patients received other treatments (radiation or concurrent chemoradiation therapy) before surgery; (2) The articles were case reports, reviews, meta-analysis, organizational guidelines, letters, expert opinions, or conference abstracts; (3) The studies had inadequate data for outcome assessment; (4) The articles had no outcomes of interest. (5) The published Articles were not in English.

### 2.3. Data extraction and quality assessment

Data were extracted into a standard form, and included information on the first author, publication year, country, participants’ characteristics, study design, number of study participants, surgical approaches, and FIGO stage. Primary outcomes were intraoperative total complications and postoperative aggregate complication. Secondary outcomes were categorized into two groups (individual intraoperative and postoperative complications). Individual intraoperative complications included bladder damage, cystotomy, bowel injury, subcutaneous emphysema, nerve injury, ureteral injury, and vessel injury. Postoperative complications included wound infection, incisional hernia, pelvic infection and abscess, lymphedema, lymphocyst, intestinal obstruction, pulmonary embolism, deep vein thrombosis, and fistula. In this meta-analysis, we used the Newcastle-Ottawa scale to evaluate 39 studies and the Jadad scale to evaluate 1 study S2 and S3 Tables [14, 15]. Two reviewers independently evaluated and cross-checked the qualities of the included studies, as well as assessed the bias of the studies. Disagreements were discussed between two evaluators in order to reach a consensus and the third reviewer also provided the opinion.

### 2.4. Data synthesis and meta-analysis

This meta-analysis was conducted using Stata SE version 12.0 software (StataCorp, College Station, TX). We analyzed heterogeneity with the chi-square test, and P-value < 0.10 was used to establish statistical significance with I^2^ test [16]. I^2^ values > 50% were considered substantial evidence of statistical heterogeneity. To estimate pooled odds ratio (OR) with 95% confidence interval (CI), a fixed-effects model was used in the absence of significant heterogeneity; the random-effects model was used in the presence of significant heterogeneity [17]. We evaluated the publication bias for each of the pooled study groups with a funnel plot. We carried out subgroup analysis based on the modalities of MIS (LAVRH, total laparoscopic radical hysterectomy (TLRH), and RRH) to assess the outcomes of different subgroups.

## 3. Results

**Fig 1:**
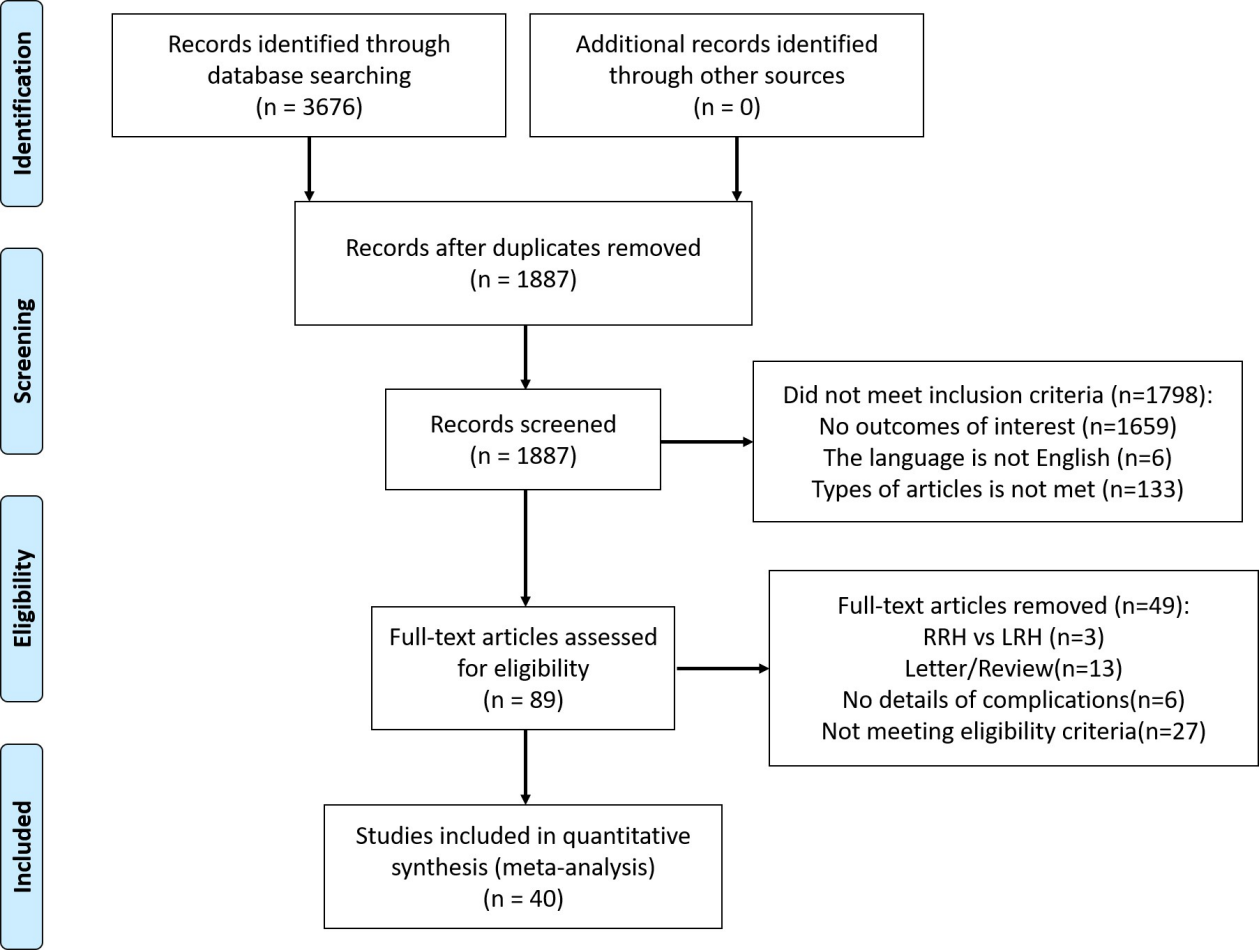
Flow chart of study selection in this meta-analysis.

A total of 40 studies were included in this analysis. The flowchart of the selection process is shown in Fig 1. The initial search retrieved 3,673 articles from the four databases. All articles were imported into Endnote for screening. After excluding duplicates, 1,887 articles were identified for the next step of screening. By reviewing titles and abstracts, 1,798 articles were removed for not meeting the selection criteria, and 89 articles were identified to be assessed for eligibility. Eventually, 40 studies were identified in the final analysis, and all of them were screened after reviewing the full text. We used the Newcastle-Ottawa scale to assess the quality of 39 studies and Jadad scale to assess 1 RCT, Table 1 shows the results of included studies.

**Table 1:** Characteristics of the 40 studies included in the meta-analysis.

Study cohort	Year	Country	Study design	Approach	Number(N)	FIGO stage (N)	BMI^a^	Age^a^(years)	Score^b^
Lee et al.	2002	China, Taiwan	prospective	LAVRH	30	IA-IB 30	54.4 ± 12.6	46.2(32–64)	6
				ORH	30	IA-IB 30	56.3 ± 10.4	48.0(34–68)	
Steed et al.	2004	Canada	retrospective	LAVRH	71	IA-IB 71	-	43 (30–69)	6
				ORH	205	IA-IB 205	-	44 (24–86)	
Sharma et al.	2006	England	retrospective	LAVRH	35	IA2–IIB 35	-	43.4(28–60)	8
				ORH	32	IA2–IIB 32	-	42.8(28–66)	
Frumovitz et al.	2007	USA	retrospective	LRH	35	IA-IB 35	28.1(18.4–40.8)	40.8(28.4–63.4)	8
				ORH	54	IA-IB 54	28.2(17.4–46.4)	42.5(27.3–68.3)	
Li et al.	2007	China	retrospective	LRH	90	IB-IIA 90	-	42 ± 9	6
				ORH	35	IB-IIA 35	-	44 ± 11	
Morgan et al.	2007	Ireland	retrospective matched	LAVRH	30	IA–IB 30	25 (18.6–47)	35 (25–54)	6
				ORH	30	IA–IIB 30	24 (19.8–29.5)	38 (20–63)	
Uccella et al.	2007	Italy	retrospective	LRH	50	IA2–IIA 50	23 (17.4–35)	47 (24–78)	7
				ORH	48	IA2–IIA 48	25 (19–43)	53 (28–75)	
Zakashansky et al.	2007	USA	retrospective matched	LRH	30	IA1–IIA 30	-	48.3 ± 12.25	7
				ORH	30	IA1–IIA 30	-	46.6 ± 11.75	
Boggess et al.^c^	2008	USA	retrospective	RRH	51	IA1–IIA 47	28.6 ± 7.2	47.4 ± 12.9	6
				ORH	49	IA2–IIA 49	26.1 ± 5.1	41.9 ± 11.2	
Ko et al.	2008	USA	retrospective	RRH	16	IA1–IB1 16	27.6 ± 6.4	42.3 ± 7.9	5
				ORH	32	IA1–IIA 32	26.6 ± 5.9	41.7 ± 8.1	
Estape et al.	2009	USA	retrospective	RRH	32	IB1-IB2 32	29.7 ± 3.2	55.0(33–83)	7
				LRH	17	IA2-IB2 17	28.1 ± 4.8	52.8(37–83)	
				ORH	14	IB1-IB2 14	29.5 ± 6.4	42.0(27–71)	
Maggioni et al.	2009	USA	retrospective	RRH	40	IA2–IIA 40	24.1 ± 5.5	44.1 ± 9.1	7
				ORH	40	IA2–IIA 40	23.6 ± 5.0	49.8 ± 14.1	
Malzoni et al.	2009	Italy	retrospective	TLRH	65	IA1–IB1 65	26(19–35)	40.5 ± 7.7	9
				ORH	62	IA1–IB1 62	29(19–35)	42.7 ± 8.6	
Papacharalabous et al.	2009	UK	retrospective	LAVRH	14	IA2–IB 14	-	38.6 ± 3.6	8
				ORH	12	IA2–IB 12	-	43.5 ± 12.9	
Sobiczewski et al.	2009	Poland	retrospective	LRH	22	IA1–IB1 22	-	45.44 ± 9	8
				ORH	58	IA1–IIA 58	-	51.19 ± 12	
Schreuder et al. ^c^	2010	Netherlands	retrospective	RRH	13	IB1-IIB 13	-	43 (31–78)	7
				ORH	14	IB1-IB2 14	-	46 (32–68)	
Lee et al.	2011	ROK	retrospective	LRH	24	IA2–IIa 24	23.4±3.55	48.4 ± 7.25	9
				ORH	48	IA2–IIa 48	23.9±4.7	50.2 ± 8.25	
Sert et al.	2011	Norway	retrospective	RRH	35	IA1–IB1 35	25.4±4.36	44.1 ± 10.5	9
				LRH	7	IA1–IB1 7	22.5±1.84	45.0 ± 12.9	
				ORH	26	IA1–IB1 26	25±3.0	44.8 ± 11.8	
Taylor et al.	2011	USA	retrospective	LAVRH	9	IA2–IB1 9	26.3 (20.6–36.1)	41.4 (31–60)	7
				ORH	18	IA2–IB1 18	26.9 (17–38.3)	41.1 (25–61)	
Gortchev et al.	2012	Bulgaria	retrospective	RRH	73	-	-	46.0 ± 11.2	8
				LAVRH	46	-	-	42.5 ± 9.9	
				ORH	175	-	-	49.0 ± 11.0	
Nam et al.	2012	Korea	retrospective matched	LRH	263	IA2–IIA 263	-	-	8
				ORH	263	IA2–IIA 263	-	-	
Park et al.	2012	Korea	retrospective	LRH	54	IA2–IIA2 54	31.8 ± 1.39	49.4 ± 11.5	7
				ORH	112	IA2–IIA2 112	31.7 ± 1.5	52.1 ± 11.8	
Lim et al.	2013	Singapore	prospective	LRH	18	IA1-IIA 18	22.9 (16.0–33.7)	48 (30–65)	9
				ORH	30	IA1-IIA 30	22.4 (17.9–33.9)	47 (33–67)	
Park et al.	2013	Korea	retrospective	LRH	115	IB2-IIA2 115	23.1 (15.6–34.8)	48.5 (25–77)	8
				ORH	188	IB2-IIA2 188	23.7 (17.6–34.7)	48.1 (25–84)	
BoganI et al.	2014	Italy	retrospective	LRH	65	IA2-IIB 65	25.1 ± 5.2	48.9 ±13.5	9
				ORH	65	IA2-IIB 65	25.9 ± 6.1	50.9 ± 14	
Chen et al.	2014	Taiwan	retrospective	RRH	24	IA-IIB 24	24.4 ± 4.9	53.7 ± 15.3	8
				LRH	32	IA-IIB 32	23.2 ± 3.4	51.2 ± 11.9	
				ORH	44	IA-IIB 44	24.9 ± 4.6	51.9 ± 11.3	
Yin et al.	2014	China	retrospective	LRH	22	IA2–IIA 22	-	44 ± 1.5	6
				ORH	23	IA2–IIA 23	-	46 ± 2.3	
Asciutto et al.	2015	Sweden	retrospective	RRH	64	IA2–IIA 64	27.0 ± 6.1	45.4 ± 13.6	6
				ORH	185	IA2–IIA 178	25.7 ± 4.7	45.7 ± 13.0	
Ditto et al.	2015	Italy	retrospective matched	LRH	60	IA2–IB1 60	24.3 ± 2.9	46 (29–79)	9
				ORH	60	IA2–IB1 60	24.0 ± 4.3	45.5 (15–78)	
Xiao et al.	2015	China	retrospective	LRH	106	IA-IIB 106	23.8 ± 3.9	43.7 ± 9.3	8
				ORH	48	IA-IIB 48	24.7 ± 3.8	45.7 ± 11.3	
Park et al.	2016	Korea	retrospective	LRH	186	IA2–IIA1 186	23.69 (17.1–34.9)	45.3 (27–71)	7
				ORH	107	IA2–IIA1 107	23.58 (17.1–35.9)	47.3 (28–73)	
Shah et al.	2017	USA	retrospective	RRH	109	IA1-IB2 109	27.9 (17.6–51.6)	45.2 (25–84)	7
				ORH	202	IA1-IB2 202	29.1 (18.3–55.7)	45.4 (19–88)	
Corrado et al.	2018	Italy	retrospective	RRH	88	IB1 88	23.3 (18–47.6)	46 (27–77)	8
				LRH	152	IB1 152	23.5 (17–35)	45 (23–78)	
				ORH	101	IB1 101	24.8 (18–51)	50 (28–76)	
Guo et al.	2018	China	retrospective	LRH	412	IA-IIA 412	22.81 (14.3–35.6)	44.19 (25–76)	7
				ORH	139	IA-IIA 139	23.19 (13.8–36.6)	40.52 (23–62)	
Bogani et al. ^c^	2019	Italy	Retrospective matched	LRH	35	IB1-IIA 23	22.9 ± 4.0	41.1 ± 6.9	7
				ORH	35	IB1-IIA 24	20.1 ± 9.3	44.1 ± 12.7	
Matanes et al.	2019	Israel	retrospective	RRH	74	IA1-IIA 74	26.4(18.2–42.1)	48(29–77)	8
				ORH	24	IA1-IIA 24	26.2(20.6–38.5)	47(24–69)	
Piedimonte et al.	2019	Canada	Retrospective	RRH	749	-	-	-	6
				ORH	2584	-	-	-	
Yuan et al.	2019	China	Retrospective matched	LRH	99	IIA2-IIA2 99	44.56 ± 7.60	43.58 ± 8.86	9
				ORH	99	IIA2-IIA2 99	24.56 ± 1.50	44.56 ± 7.60	
Pahisa et al.	2010	Spain	Retrospective	LAVRH	67	IA2-IIA 67	25.4 ± 1.1	51 (29–75)	7
				ORH	23	IA2-IIA 23	27.2 ± 2.5	48 (31–67)	
Campos et al.	2013	Brazil	RCT	LRH	16	IA2–IB 16	-	36.19 ± 9.78	5
				ORH	14	IA2–IB 14	-	39.64 ± 6.23	

ORH: Open radical hysterectomy, LRH: Laparoscopic radical hysterectomy, RRH: Robotic radical hysterectomy, LAVRH: Laparoscopic-assisted vaginal radical hysterectomy, RCT: Randomized controlled trial

a: Mean, median or unknow.

b: Jadad scale: score: 1~3, indicating low quality study; score: 4~7, indicating high quality study. Newcastle-Ottawa scale: score≤5, indicating high risk of bias; score>5, indicating low risk of bias.

c: These studies including other FIGO stages of cervical cancer.

The main characteristics of the 40 studies are shown in Table 1. The study designs were as follow: retrospective study (n = 31) [18–48], retrospective matched study (n = 6) [49–54], prospective cohort study (n = 2) [55, 56], and RCT (n = 1) [57]. Thirteen studies were conducted in Asia (China, Israel, Korea, Singapore, and Taiwan) [21, 35, 36, 38, 39, 41, 42, 45, 47, 52, 54–56], ten in North America (Canada, and USA) [18, 20, 23–26, 33, 43, 48, 51], sixteen in Europe (UK, Ireland, Poland, Netherlands, Federal Republic of Germany, Norway, Bulgaria, Italy, Sweden, and Spain) [19, 22, 27–32, 34, 37, 40, 44, 46, 49, 50, 53], and one study in South America (Brazil) [57]. In all, we identified 9003 patients in the pooled analysis: 2277 patients had LRH, 1,368 patients had RRH and 5358 patients had ORH (we compared 1,368 patients who underwent RRH vs 3,490 patients who underwent ORH, and 2277 patients who underwent LRH vs 2,228 patients who underwent ORH). As shown in Table 1, 8 studies compared RRH with ORH [23, 24, 26, 29, 39, 42, 46, 47], 25 studies compared LRH with ORH [18–22, 27, 28, 30, 32, 34–36, 38, 40, 41, 44, 45, 48–57], and 5 studies compared all 3 surgical approaches [25, 31, 33, 37, 43].

### 3.1 MIS vs ORH

#### 3.1.1 Primary outcomes

**Fig 2:**
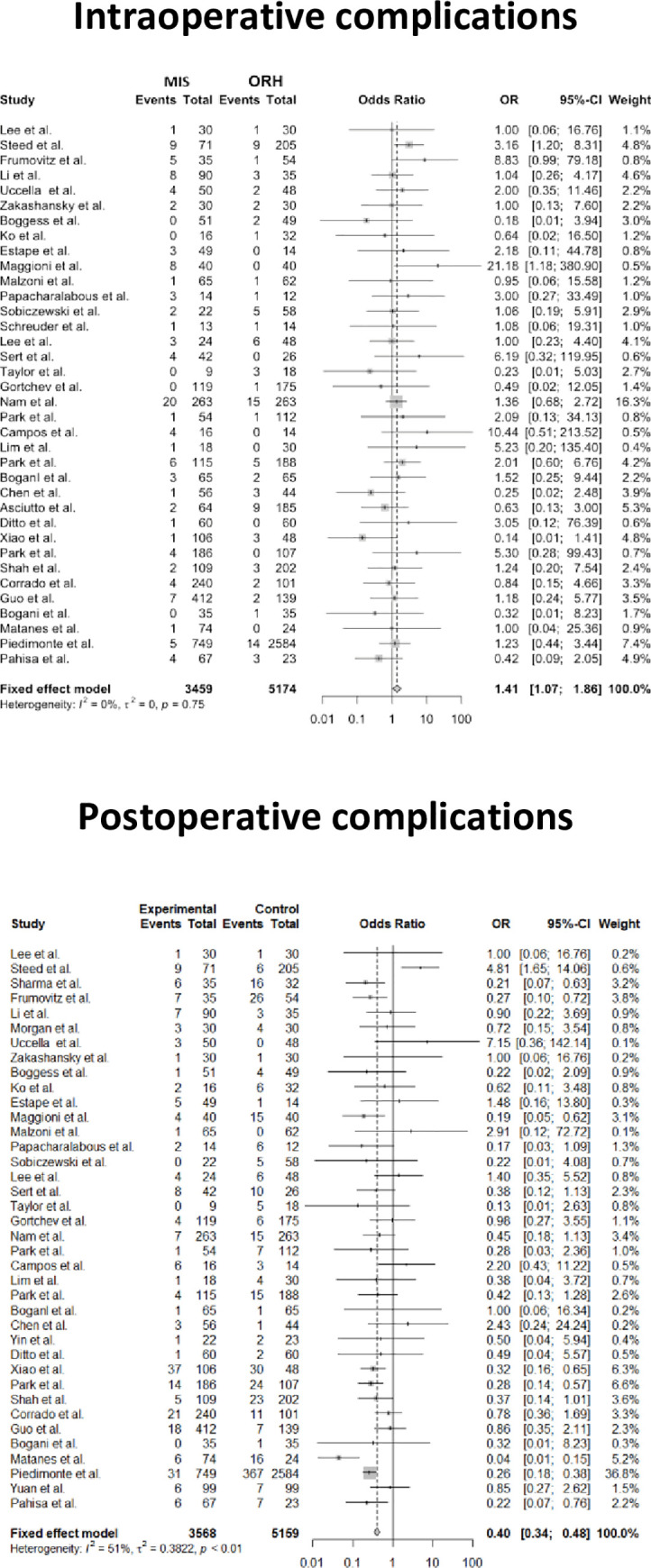
Forest plots of intraoperative and postoperative complications between Minimally Invasive Surgical (MIS) and Open Radical Hysterectomy (ORH). OR, odds ratio.

**Fig 3:**
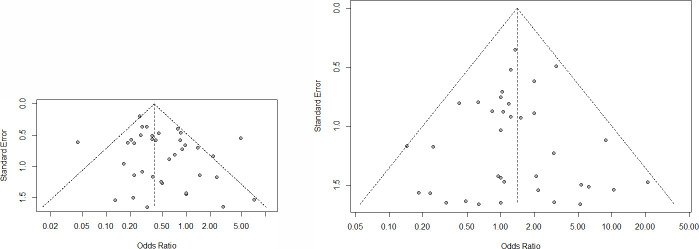
Funnel plot of studies evaluating the publication bias of intraoperative and intraoperative complications between MIS and ORH. (A). intraoperative complications. (B). postoperative complications.

We show the results of intraoperative aggregate complications and postoperative overall complications between MIS and ORH in Fig 2, respectively. For intraoperative complications, the incidence of intraoperative complications in MIS (121/3459) were statistically higher than ORH (102/5174), and the risk of intraoperative complications (OR = 1.41, 95% CI = 1.07–1.86, P<0.05) in MIS was higher compared with ORH. In terms of postoperative complications, MIS was associated with significantly lower risk of postoperative complications (OR = 0.40, 95% CI = 0.34–0.48, P = 0.0143) compared with ORH. There was no heterogeneity in studies of intraoperative aggregate complications (I^2^ = 0%, P = 0.748). However, we found that the studies of postoperative overall complications were associated with high heterogeneity (I^2^ = 51%, P<0.01). The result of publication bias was shown in Fig 3, the funnel plot was nearly symmetric on both sides, so there was no publication bias in the results of intraoperative aggregate complications and postoperative overall complications.

#### 3.1.2 Secondary outcomes

In order to determine the source of difference, we analyzed the results of individual intraoperative and postoperative complications in Table 2, respectively. For intraoperative complications, there were no significant differences between MIS and ORH in the bladder damage, nerve injury, ureteral injury, or vessel injury, with ORs of 1.28 (95% CI = 0.75–2.19, P = 0.3), 0.51 (95% CI = 0.14–1.93, P = 0.343), 1.05 (95% CI = 0.61–1.76, P = 0.959), 1.01 (95% CI = 0.59–1.73, P = 0.753), respectively. However, MIS was associated with increased risk of cystotomy (OR = 2.27, 95% CI = 1.23–4.20), bowel injury (OR = 2.15, 95% CI = 0.95–4.89), subcutaneous emphysema (OR = 4.36, 95% CI = 0.94–20.29) in comparison with ORH. In terms of postoperative complications, there were comparable in the risk of incisional hernia (OR = 0.93, 95% CI = 0.34–2.51, P = 0.803) and lymphocyst (OR = 0.73, 95% CI = 0.46–1.15, P = 0.123) between MIS and ORH. Comparing to ORH, MIS was associated with significantly lower risks of wound-infection (OR = 0.15, 95% CI = 0.08–0.28, P<0.01), pelvic infection and abscess (OR = 0.40, 95% CI = 0.26–0.63, P<0.01), lymphedema (OR = 0.48, 95% CI = 0.24–0.98, P = 0.03), intestinal obstruction (OR = 0.30, 95% CI = 0.21–0.43, P<0.01), pulmonary embolism (OR = 0.36, 95% CI = 0.09–1.48, P = 0.025), deep vein thrombosis (OR = 0.56, 95% CI = 0.35–0.88, P = 0.01), and urinary tract infection (OR = 0.56, 95% CI = 0.34–0.91, P = 0.013). However, the risk of fistula (OR = 1.69, 95% CI = 0.02–2.79, P = 0.011) was significant increased in the MIS group than in ORH.

**Table 2:** Meta-analysis estimates of individual complications between MIS and ORH.

Category	MIS	ORH	OR (95% CI)	P value	I^2^(%)
Transfusion	301/2490	494/4408	0.34[0.22,0.53]	**<0.001**	72.3
**Intraoperative complications**					
Bladder damage	25/2279	24/4009	1.28[0.75,2.19]	0.3	0
Cystotomy	32/586	14/677	2.27[1.23,4.20]	**0.002**	0
Bowel injury	12/1479	8/3449	2.15[0.95,4.89]	**0.041**	0
Subcutaneous emphysema	7/246	0/207	4.36[0.94,20.29]	**0.008**	0
Nerve injury	2/1181	5/802	0.51[0.14,1.93]	0.343	0
Ureteral injury	22/2519	24/4520	1.05[0.61,1.76]	0.959	0
Vessel injury	21/2328	27/4112	1.01[0.59,1.73]	0.753	0
**Postoperative complications**					
Wound infection	5/1380	104/3277	0.15[0.08,0.28]	**<0.001**	0
Incisional hernia	7/898	7/811	0.93[0.34,2.51]	0.803	0
Pelvic infection and abscess	30/1713	78/3396	0.40[0.26,0.63]	**<0.001**	39.9
Lymphedema	13/791	19/619	0.48[0.24,0.98]	**0.03**	0
Lymphocyst	40/1614	35/1194	0.73[0.46,1.15]	0.123	8.4
Intestinal obstruction	37/2490	281/4070	0.30[0.21,0.43]	**<0.001**	0
Pulmonary embolism	0/508	7/558	0.36[0.09,1.48]	**0.025**	0
Deep vein thrombosis	31/2289	78/3886	0.56[0.35,0.88]	**0.01**	0
Fistula	38/2203	17/1904	1.69[0.02,2.79]	**0.011**	0
Urinary tract infection	33/764	44/799	0.56[0.34,0.91]	**0.013**	3

**OR: Odds ratio; CI: Confidence interval; MIS: Minimally invasive surgery; ORH: Open radical hysterectomy**

### 3.2 Subgroup analysis

The subgroup analysis compared intraoperative complications and postoperative complications among the three types of MIS, as shown in Table 3. For intraoperative aggregate complications, compared to ORH, the risks of complications were not increased in RRH (OR = 1.11, 95% CI = 0.62–2.01, P = 0.11) and TLRH (OR = 1.34, 95%CI = 0.94–1.93, P = 0.722), whereas it was higher in LAVRH (OR = 2.27, 95%CI = 1.02–5.04, P = 0.044). For postoperative overall complication, the risk in LAVRH (OR = 0.71, 95%CI = 0.26–1.93, P = 0.506) was not statistically different from that of ORH. However, RRH (OR = 0.42, 95%CI = 0.26–0.68, P<0.01) and TLRH (OR = 0.58, 95%CI = 0.45–0.74, P<0.01) was associated with a reduced risk of postoperative complication when compared with ORH. In a stratified analysis (S4 Table), in an attempt to further determine the difference in fistula complications, we also analyzed complications with different types of fistula, including vesicovaginal, rectovaginal, ureterovaginal and urinary fistula, with ORs of 1.55 (95%CI = 0.59–4.06, P = 0.376), 2.88 (95%CI = 0.44–18.70, P = 0.269), 1.60 (95%CI = 0.59–4.34, P = 0.353), and 1.25 (95%CI = 0.53–2.97, P = 0.612) respectively. Interestingly, there was no significant difference in risk of the individual fistula types between MIS and ORH.

**Table 3:** The subgroup analysis of laparoscopic types between MIS and ORH in intraoperative and postoperative overall complications.

Category	Laparoscopic type	Study	OR (95% CI)	P value	I^2^(%)
**Intraoperative complications**					
	TLRH	23	1.34[0.94,1.93]	0.11	0
	LAVRH	5	2.27[1.02,5.04]	**0.044**	0
	RRH	13	1.11[0.62,2.01]	0.722	0
**Postoperative complications**					
	TLRH	25	0.58[0.45,0.74]	**<0.01**	0
	LAVRH	7	0.71[0.26,1.93]	0.506	58.5
	RRH	11	0.42[0.26,0.68]	**<0.01**	45.3

**OR: Odds ratio; CI: Confidence interval; TLRH: Total laparoscopic radical hysterectomy; RRH: Robotic radical hysterectomy; LARVH: Laparoscopic assisted radical vaginal hysterectomy.**

## 4. Discussion

This study assessed most comprehensive results of complications of cervical cancer surgeries and evaluated the safety of different surgical strategies. The rates of perioperative complications will become a key factor of importance in comparing surgical modalities for managing cervical cancer. We aimed to provide a basis for the selection of optimal surgical methods, as well as offer new opinions for actual role of MIS in cervical cancer.

Our meta-analysis indicated that the overall risk of intraoperative complications was increased with MIS than with ORH. Patients accepted to MIS experienced almost 2 times the risk of intraoperative complications compared with patients accepted to ORH. There were no significant differences in risk for intraoperative complications including bladder damage, nerve injury, ureteral injury, and vessel injury among individual intraoperative complications. However, MIS group was associated with higher risk in complications of cystotomy, bowel injury, and subcutaneous emphysema in comparison to ORH. This finding was consistent with previous studies. The differences in bowel injury between MIS and ORH can be explained by the use of surgical instruments such as a trocar and Veress needle during radical hysterectomy. Previous studies have shown that the majority of bowel injuries occurred during laparoscopy using a Veress needle or trocar placement [58, 59]. The subcutaneous emphysema was the unique complications in MIS, many risk factors will lead to it during MIS including increased intra-abdominal pressure, total gas volume, and gas flow rate [60].

Regarding postoperative complications, our meta-analysis found that MIS was associated with significantly lower risk of postoperative overall complications compared with ORH. In individual postoperative complications, incisional hernia and lymphocyst had no differences between MIS and ORH. MIS was superior to ORH in terms of wound infection, pelvic infection and abscess, lymphedema, intestinal obstruction, pulmonary embolism, and urinary tract infection, whereas the risk of fistula complications was significantly increased, with MIS compared to ORH. Interestingly, in a stratified analysis of fistula complications, we found that there were no significant differences in risk for four types of fistula complications. Possible reasons for this result including individual fistula complication had small sample size and excessive weight of included studies biased the results [61]. Although there were no significant differences in the risks of vesicovaginal, rectovaginal, ureterovaginal, and urinary fistula between MIS and ORH, the incidence rates of these four types of fistula complications in MIS were higher than that of ORH. This finding was worthy of our attention.

Taken together, the surgeon proficiency may be a factor in determining the rates of complications. Regrettably, this meta-analysis was not able to provide a comparison between surgeons. Furthermore, the learning curve could play an important role in complications between different surgical modalities, and MIS was associated with a longer learning curve than ORH because of the complexity of surgical procedure, and also might have influenced complication rates [62, 63]. The use of surgical instruments was related to viscus injuries, which may be caused by thermal injury, due to the high temperature of the surgical instruments resulting in the damage of submucosal or deeper tissues of the bladder, intestines, and bowel. Previous studies have evaluated the thermal injury of bowel in laparoscopic approach [62]. It must be taken that thermal injury was an inherent risk of the technique during radical hysterectomy, and therefore surgeon should pay attention to this issue. Overall, these factors were associated with the incidence of intraoperative and postoperative complications.

Concerning the subgroup meta-analysis of surgical modalities, intraoperative complication rate increased in the course of LAVRH, as well as there were no differences in TLRH and RRH. This finding is consistent with that of previous meta-analyses. The requirement for refinement of LAVRH is very high due to the complex pelvic floor anatomy in females. In the vaginal approach, the ureters and bladder are identified by traction on the uterus after the ligament around the uterus is isolated and cut [64], and urinary tract trauma is a clear risk during LAVRH. With time, laparoscopy is continually evolving with the improvement in surgical skills, instruments, and learning curve, and these improvements may be partly responsible for reduction in intraoperative complication over time [63]. For postoperative aggregate complications, both RRH and TLRH were associated with lower risk compared to ORH. These results were validated in previous studies, Park et al. compared the complications of three approaches, RRH had a positive effect in reducing overall complications than ORH for cervical cancer patients [65]. For LAVRH group, the high heterogeneity and the small sample size could bias the results of postoperative complications. In the future, we need more high-quality cohort studies to evaluate and compare the risk of postoperative complications between MIS and ORH.

There are limitations to this meta-analysis. First, included studies were primarily non-randomized studies, which could not provide high-quality evidence. Furthermore, our study did not include single-arm studies, which can lead to the bias of the result. Additionally, differences in patients’ characteristics between different surgical cohorts may lead to highly heterogeneous outcomes in studies and affect the results of the pooled analysis. The statistical methods could not fully diminish these differences. Second, the difference of surgeons in these articles were not reported including the level of experience in surgeons and types of surgeons, these factors could affect the surgical outcomes as time went by. The additional morbidities of patients in these studies were not involved, these factors could contribute to the bias of results. Third, most studies included in this meta-analysis did not use standardized methods of classifying complications, such as the Clavien-Dindo classification system, and the final results may be affected by these differences in the reporting of complications. Among all included studies, only one adopted the Clavien-Dindo classification system of complications [39]. Forth, during the extraction of complication data, many studies revealed that patients had undergone cesarean section or previous abdominal surgery and had severe adhesions in the past, alluding to the fact that the success of laparoscopy will be affected by adhesions. Therefore, the incidence of complications ultimately may interfere with the results and may be a cause of bias.

## 5. Conclusion

Our meta-analysis demonstrates that MIS is superior to laparotomy, with fewer postoperative overall complications (wound infection, pelvic infection and abscess, lymphedema, intestinal obstruction, pulmonary embolism, and urinary tract infection). However, MIS is associated with a higher risk of intraoperative aggregate complications (cystotomy, bowel injury, and subcutaneous emphysema) and postoperative fistula complications. In the future, high-quality prospective studies and RCTs are needed to provide sufficient evidence for evaluating the pros and cons of using MIS to treat cervical cancer.

## Supporting information

S1 Checklist PRISMA 2009 checklist. (DOC)

S1 Fig PRISMA 2009 flow diagram. (DOC)

S1 Table Detailed search strategy. (DOC)

S2 Table Quality assessment of the included studies according to modified NOS score. (DOC)

S3 Table Quality assessment of the included studies according to modified Jadad score. (DOC)

S4 Table The subgroup analysis of fistula types between MIS and ORH. (DOC)
