# Supplementary material for: Comparison of the complications between minimally invasive surgery and open surgical treatments for early-stage cervical cancer: A systematic review and meta-analysis
Source: PLoS One. 2021 Jul 1;16(7):e0253143. doi: 10.1371/journal.pone.0253143 (PMC8248723; doi:10.1371/journal.pone.0253143)
Supplement: S1 Fig — (DOC) [file pone.0253143.s002.doc]

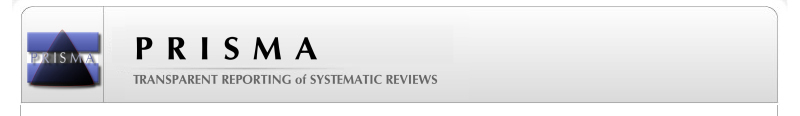
**S1 Fig. PRISMA 2009 Flow Diagram**

**Screening**

**Included**

**Eligibility**

**Identification**

Records identified through database searching
(n = 3676 )

Additional records identified through other sources
(n = 0 )

Records after duplicates removed
(n = 1887 )

Records screened
(n = 1887 )

Did not meet inclusion criteria (n=1798):
No outcomes of interest (n=1659)

The language is not English (n=6)

Types of articles is not met (n=133)

Full-text articles assessed for eligibility
(n = 89 )

Full-text articles removed (n=49):

RRH vs LRH (n=3)

Letter/Review(n=13)

No details of complications(n=6)

Not meeting eligibility criteria(n=27)

Studies included in quantitative synthesis (meta-analysis)
(n = 40 )
