# Supplementary material for: Comparison of the complications between minimally invasive surgery and open surgical treatments for early-stage cervical cancer: A systematic review and meta-analysis
Source: PLoS One. 2021 Jul 1;16(7):e0253143. doi: 10.1371/journal.pone.0253143 (PMC8248723; doi:10.1371/journal.pone.0253143)
Supplement: S1 Table — (DOC) [file pone.0253143.s003.doc]

| #1 Open Surgery |
| --- |
| #2 Open Surgical Procedure |
| #3 Open Procedure |
| #4 Laparotom* |
| #5 1 or 2 or 3 or 4 |
| #7 Minimal* Invasive Surg* |
| #8 Minimal* Access Surg* |
| #9 Minimal* Invasive Surg* Procedure* |
| #10 Minimal* Surg* Procedure* |
| #12 7 or 8 or 9 or 10 |
| #13 Robot* Surg* Procedure* |
| #14 Robot-Enhanced Procedure* |
| #15 Robot-Enhanced Surg* |
| #16 13 or 14 or15 |
| #17 Laparoscop* |
| #18 Celioscop* |
| #19 Laparoscop* Surg* |
| #20 17 or 18 or 19 |
| #21 12 or 16 or 20 |
| #22 5 and 21 |
| #23 Uterine Cervical Neoplasm* |
| #24 Cervi* Cancer* |
| #25 Cervical Neoplasm* |
| #26 23 or 24 or 25 |
| Combined:835 |

**S1 Table. Detailed search strategy.**

**PubMed search 2020.02.21**

**All individual searches from each database inception date until 20 February 2020**

**Embase search 2020.02.21**

| #1 cervix* AND adj3 |
| --- |
| #2 cancer* |
| #3 tumor* |
| #4 tumour* |
| #5 neoplas* |
| #6 carcinoma* |
| #7 malignan* |
| #8 uterine cervix tumor/exp |
| #9 2 or 3 or 4 or 5 or 6 or 7 |
| #10 1 and 9 |
| #11 8 and 10 |
| #12 endoscop* OR laparotom* OR laparoscop* OR celioscop* OR peritoneoscop* |
| #13 Minimal* (Surg* or Procedure*) |
| #14 laparotom* OR (open AND surg*) |
| #15 Robot* AND Surg* |
| #17 12 or 13 or 14 |
| #18 11 and 17 |
| Combined:1832 |

**All individual searches from each database inception date until 20 February 2020**

**Cochrane search 2020.02.21**

| #1 uterine cervix tumor |
| --- |
| #2 cervix* adj3 (cancer* OR tumor* OR tumour* OR neoplas* OR carcinoma* OR malignan* ) |
| #3 1 or 2 |
| #4 laparotomy OR laparotom* OR (open AND surger*) |
| #5 minimal* AND (surgi* OR procedure*) |
| #6 endoscop* OR laparotom* OR laparoscop* OR celioscop* OR peritoneoscop* |
| #7 5 or 6 |
| #8 Robot* AND Surg* |
| #9 4 or 7 or 8 |
| #9 3 and 9 |
| Combined:70 |

**All individual searches from each database inception date until 20 February 2020**

**Web of science search 2020.02.21**

| #1 Uterine Cervical Neoplasm* |
| --- |
| #2 Cervi* Cancer* |
| #3 Cervical Neoplasm* |
| #4 1 or 2 or 3 |
| #5 laparotomy OR laparotom* OR (open AND surger*) |
| #6 Minimal* AND (surgi* OR procedure*) |
| #7 endoscop* OR laparotom* OR laparoscop* OR celioscop* OR peritoneoscop* |
| #8 6 or 7 |
| #9 Robot* AND Surg* |
| #10 5 or 8 or 9 |
| #11 4 and 10 |
| Combined:939 |

**All individual searches from each database inception date until 20 February 2020**
