# Supplementary material for: Comparison of the complications between minimally invasive surgery and open surgical treatments for early-stage cervical cancer: A systematic review and meta-analysis
Source: PLoS One. 2021 Jul 1;16(7):e0253143. doi: 10.1371/journal.pone.0253143 (PMC8248723; doi:10.1371/journal.pone.0253143)
Supplement: S2 Table — (DOC) [file pone.0253143.s004.doc]

**S2 Table. Quality Assessment of the Included Studies According to Modified NOS score.**

| **Study** | **Quality indicators from Newcastle-Ottawa scale** | | | | | | | | | | **Score** |
| --- | --- | --- | --- | --- | --- | --- | --- | --- | --- | --- | --- |
|  | | **Selection** | | | | **Comparability** | | **Exposure/outcome** | | |  |
|  | | 1 | 2 | 3 | 4 | 5a | 5b | 6 | 7 | 8 |  |
| Lee et al. | | Yes | Yes | Yes | Yes | No | No | Yes | Yes | No | 6 |
| Steed et al. | | Yes | Yes | Yes | Yes | Yes | No | No | Yes | Yes | 6 |
| Sharma et al. | | Yes | Yes | Yes | Yes | Yes | No | Yes | Yes | Yes | 8 |
| Frumovitz et al. | | Yes | Yes | Yes | Yes | Yes | Yes | No | No | Yes | 8 |
| Li et al. | | Yes | Yes | Yes | Yes | Yes | No | Yes | No | No | 6 |
| Morgan et al. | | Yes | Yes | Yes | Yes | Yes | No | Yes | No | No | 6 |
| Uccella et al. | | Yes | Yes | Yes | Yes | Yes | Yes | Yes | No | No | 7 |
| Zakashansky et al. | | Yes | Yes | Yes | Yes | Yes | Yes | Yes | No | No | 7 |
| Boggess et al. | | Yes | Yes | Yes | Yes | Yes | No | Yes | No | No | 6 |
| Ko et al. | | Yes | Yes | Yes | Yes | No | No | Yes | No | No | 5 |
| Estape et al. | | Yes | Yes | Yes | Yes | No | No | Yes | Yes | Yes | 7 |
| Maggioni et al. | | Yes | Yes | Yes | Yes | Yes | No | Yes | Yes | No | 7 |
| Malzoni et al. | | Yes | Yes | Yes | Yes | Yes | Yes | Yes | Yes | Yes | 9 |
| Papacharalabous et al. | | Yes | Yes | Yes | Yes | Yes | No | Yes | Yes | Yes | 8 |
| Sobiczewski et al. | | Yes | Yes | Yes | Yes | Yes | No | Yes | Yes | Yes | 8 |
| Schreuder et al. | | Yes | Yes | Yes | Yes | No | No | Yes | Yes | Yes | 7 |
| Lee et al. | | Yes | Yes | Yes | Yes | Yes | Yes | Yes | Yes | Yes | 9 |
| Sert et al. | | Yes | Yes | Yes | Yes | Yes | Yes | Yes | Yes | Yes | 9 |
| Taylor et al. | | Yes | Yes | Yes | Yes | No | No | Yes | Yes | Yes | 7 |
| Gortchev et al. | | Yes | Yes | Yes | Yes | Yes | No | Yes | Yes | Yes | 8 |
| Nam et al. | | Yes | Yes | Yes | Yes | Yes | No | Yes | Yes | Yes | 8 |
| Park et al. | | Yes | Yes | Yes | Yes | No | No | Yes | Yes | Yes | 7 |
| Lim et al. | | Yes | Yes | Yes | Yes | Yes | Yes | Yes | Yes | Yes | 9 |
| Park et al. | | Yes | Yes | Yes | Yes | Yes | No | Yes | Yes | Yes | 8 |
| BoganI et al. | | Yes | Yes | Yes | Yes | Yes | Yes | Yes | Yes | Yes | 9 |
| Chen et al. | | Yes | Yes | Yes | Yes | Yes | Yes | Yes | No | Yes | 8 |
| Yin et al. | | Yes | Yes | Yes | Yes | Yes | No | Yes | No | No | 6 |
| Asciutto et al. | | Yes | Yes | Yes | Yes | No | No | Yes | No | No | 6 |
| Ditto et al. | | Yes | Yes | Yes | Yes | Yes | Yes | Yes | Yes | Yes | 9 |
| Xiao et al. | | Yes | Yes | Yes | Yes | Yes | No | Yes | Yes | Yes | 8 |
| Park et al. | | Yes | Yes | Yes | Yes | No | No | Yes | Yes | Yes | 7 |
| Shah et al. | | Yes | Yes | Yes | Yes | No | No | Yes | Yes | Yes | 7 |
| Corrado et al. | | Yes | Yes | Yes | Yes | Yes | No | Yes | Yes | Yes | 8 |
| Guo et al. | | Yes | Yes | Yes | Yes | No | No | Yes | Yes | Yes | 7 |
| Bogani et al. | | Yes | Yes | Yes | Yes | Yes | No | Yes | No | Yes | 7 |
| Matanes et al. | | Yes | Yes | Yes | Yes | Yes | No | Yes | Yes | Yes | 8 |
| Piedimonte et al. | | Yes | Yes | Yes | Yes | Yes | No | Yes | No | No | 6 |
| Yuan et al. | | Yes | Yes | Yes | Yes | Yes | Yes | Yes | Yes | Yes | 9 |
| Pahisa et al. | | Yes | Yes | Yes | Yes | Yes | No | Yes | No | Yes | 7 |
