# Supplementary material for: Comparison of the complications between minimally invasive surgery and open surgical treatments for early-stage cervical cancer: A systematic review and meta-analysis
Source: PLoS One. 2021 Jul 1;16(7):e0253143. doi: 10.1371/journal.pone.0253143 (PMC8248723; doi:10.1371/journal.pone.0253143)
Supplement: S3 Table — (DOC) [file pone.0253143.s005.doc]

**S3 Table. Quality Assessment of the Included Studies According to Modified Jadad score.**

| Study | Was the study described as randomized? | Was the method of randomization appropriate? | Was the study described as blinded? | Was the method of blinding appropriate? | Was there a description of withdrawals and dropouts? | Was there a clear description of the inclusion/exclusion criteria? | Was the method used to assess adverse effects described？ | Was the method of statistical analysis described? | Modified Jadad Score |
| --- | --- | --- | --- | --- | --- | --- | --- | --- | --- |
| Campos et al. | 1 | 1 | 1 | 1 | 1 | 1 | 0 | 1 | 7 |
