# Supplementary material for: Comparison of the complications between minimally invasive surgery and open surgical treatments for early-stage cervical cancer: A systematic review and meta-analysis
Source: PLoS One. 2021 Jul 1;16(7):e0253143. doi: 10.1371/journal.pone.0253143 (PMC8248723; doi:10.1371/journal.pone.0253143)
Supplement: S4 Table — (DOC) [file pone.0253143.s006.doc]

| **S4 Table. The subgroup analysis of fistula types between MIS and ORH** | | | | | | |  |  |
| --- | --- | --- | --- | --- | --- | --- | --- | --- |
| **Category** |  | **Fistula type** | **Study** | **MIS** | **ORH** | **OR(95% CI)** | **P value** | **I^2^(%)** |
| **Intraoperative complications** | | |  |  |  |  |  |  |
|  |  | Vesicovaginal fistula | 10 | 9/594 | 5/653 | 1.55[0.59,4.06] | 0.376 | 0 |
|  |  | Rectovaginal fistula | 3 | 3/145 | 0/143 | 2.88[0.44,18.70] | 0.269 | 0 |
|  |  | Ureterovaginal fistula | 10 | 9/610 | 4/756 | 1.60[0.59,4.34] | 0.353 | 0 |
|  |  | Urinary fistula | 10 | 17/1500 | 8/1144 | 1.25[0.53,2.97] | 0.612 | 0 |
