# Supplementary material for: Comparison of the complications between minimally invasive surgery and open surgical treatments for early-stage cervical cancer: A systematic review and meta-analysis
Source: PLoS One. 2021 Jul 1;16(7):e0253143. doi: 10.1371/journal.pone.0253143 (PMC8248723; doi:10.1371/journal.pone.0253143)
Supplement: Attachment — Submitted filename: Response to Reviewers.docx [file pone.0253143.s007.docx]

Dear Antonio Simone Laganà，

Thank you very much for your letter about our manuscript ‘Complications of surgical treatments for early-stage cervical cancer: a systematic review and meta-analysis’ submitted to ‘PLOS ONE’ (PONE-D-20-33497). Thank you for the comments from reviewers about our manuscript. These comments are all valuable and very helpful for revising and improving our paper, as well as the important guiding significance to us. We have checked manuscript and revised it according to your comments. We submit here the revised manuscript as well as the response for the comments. The changed words in manuscript were signed in red and the responses of reviewers in manuscript were highlighted. The comments of reviewers were signed in red and the responses were signed in blue. If you have any questions about this article, please do not hesitate to let me know.

Sincerely,

Corresponding author: Yongjun Wang

Department of Obstetrics and Gynecology, Peking University International Hospital, Beijing, 102206, China

E-mail address: [wyongjunhys@sina.com](mailto:wyongjunhys@sina.com)

**Response to Reviewer #1**

**Q1:** All the text needs a minor language revision in order to improve readability, some typos and grammatical errors.

Response: Thank you for these precious comments and suggestions. We have modified the errors of typos and grammar to improve readability of this manuscript.

**Q2:** Title. The title needs to provide more details regarding the review aim.

Response: Thank you for these precious comments and suggestions. Thank you very much for your advice, we also found that the previous title cannot provide more details to indicate the aim of this meta-analysis. The title of this manuscript was changed to **“****Comparison of the complications between minimally invasive surgery and open surgical treatments for early-stage cervical cancer: a systematic review and meta-analysis”**

**Q3:** Abstract. I would suggest improving the method section of the abstract providing the inclusion criteria following the PICO system. The present form of method is too generic.

Response: Thank you for these precious comments and suggestions. We have further modified our method section of the abstract following your advice.

**Methods：**

In this meta-analysis, the studies were abstracted that the outcomes of complications to compare MIS (laparoscopic or robot-assisted) and open radical hysterectomy in patients with early-stage (International Federation of Gynecology and Obstetrics classification stage IA1-IIA) cervical cancer. The primary outcomes were intraoperative urological complications, as well as postoperative fistula complications. Secondary outcomes included the other related complications. Two investigators independently performed the screening and data extraction. All articles that met the eligibility criteria were included in this meta-analysis.

**Q4:** Introduction. The introduction regarding the surgical approach for cervical cancer is not update.

Response: Thank you for these precious comments and suggestions. We have further revised the introduction regarding the surgical approach for cervical cancer following your advice.

**Q5:** Methods. Search strategy and study selection should be improved. Line 99 is conflicting with lines 111-112. I would suggest reporting the study selection following the PICO

Response: Thank you for these precious comments and suggestions. We have further modified our Search strategy and study selection following your advice. The sentence of Line 99 was removed and the study selection was modified by the PICO system.

**Q6:** Methods. Was a third investigator involved in the case of disagreement?

Response: Thank you for these precious comments and suggestions. **Disagreements were discussed between two evaluators in order to reach a consensus and the third reviewer also provided the opinion.**

**Q7:** Methods: How were the type of complications defined?

Response: Thank you for these precious comments and suggestions. We redefined the classification of complications as intraoperative and postoperative complications.

**Primary outcomes were total intraoperative complications and aggregate postoperative complication. Secondary outcomes were categorized into two groups (individual intraoperative complications and postoperative complications). Individual intraoperative complications included bladder damage, cystotomy, bowel injury, subcutaneous emphysema, nerve injury, ureteral injury, and vessel injury. Postoperative complications included wound infection, incisional hernia, pelvic infection and abscess, lymphedema, lymphocyst, intestinal obstruction, pulmonary embolism, deep vein thrombosis, and fistula.**

**Q8:** Results: I would suggest providing some data regarding the absolute risk difference and not only the OR. If it is not possible in results. I would suggest discussing this point in the discussion.

Response: Thank you for these precious comments and suggestions. We have analyzed the incidence of individual complications in the discussion.

**Although there were no significant differences in the risks of vesicovaginal, rectovaginal, ureterovaginal, and urinary fistula between MIS and ORH, the incidence rates of these four types of fistula complications in MIS were higher than that of ORH. This finding was worthy of our attention.**

**Q9:** Results: Data regarding the pooled cumulative incidence of complications should be provided.

Response: Thank you for these precious comments and suggestions. We have provided the pooled cumulative incidence of complications.

**Pooled analyses showed that MIS was associated with higher risk of intraoperative overall complications (OR=1.41, 95% CI=1.07-1.86, P<0.05) in comparison with ORH. However, compared to ORH, MIS was associated with significantly lower risk of postoperative aggregate complications (OR=0.40, 95% CI=0.34-0.48, P=0.0143).**

**Q10:** Results: Jadad scale in Table 1 is not reported in the study’s methods.

Response: Thank you for these precious comments and suggestions. The results of Jadad scale were shown in the S1 [Appendix](C:/Users/Curry/AppData/Local/youdao/dict/Application/8.9.0.0/resultui/html/index.html#/javascript:;).

**Quality Assessment of the Included Studies According to Modified Jadad score**

| Study | Was the study described as randomized? | Was the method of randomization appropriate? | Was the study described as blinded? | Was the method of blinding appropriate? | Was there a description of withdrawals and dropouts? | Was there a clear description of the inclusion/exclusion criteria? | Was the method used to assess adverse effects described？ | Was the method of statistical analysis described? | Modified Jadad Score |
| --- | --- | --- | --- | --- | --- | --- | --- | --- | --- |
| Campos et al. | 1 | 1 | 1 | 1 | 1 | 1 | 0 | 1 | 7 |

**Q11:** Lines 314-317. I would suggest provided references supporting the statements.

Response: Thank you for these precious comments and suggestions. Two references were provided to support our statements based on your suggestions. **Refer to: PMID: 20009907; PMID: 20009907**

**Q12:** Regarding study limitations, how complications were classified and standardized for this review should be clearly reported to allow reproducibility.

Response: Thank you for these precious comments and suggestions. This question have been done in the Q7 and we provided the excel of pooled complications of all included studies.

**Q13:** I would suggest discussing better the concept regarding the pooling of studies with completely different study designs.

Response: Thank you for these precious comments and suggestions. We have dicussed this question in the discussion.

**There are limitations to this meta-analysis. First, included studies were primarily non-randomized studies, which could not provide high-quality evidence. Furthermore, our study did not include single-arm studies, which can lead to the bias of the result. Additionally, differences in patients’ characteristics between different surgical cohorts may lead to highly heterogeneous outcomes in studies and affect the results of the pooled analysis. The statistical methods could not fully diminish these differences.**

**Q14:** The main concern regarding the current review is the background, which is completely out of the more recent literature regarding surgical approach for cervical cancer. This required extensive improvement.

Response: Thank you for these precious comments and suggestions. We have revised background in this article.

**Being the fourth most common cancer among women, it has been estimated that there are approximately 528, 000 new cases of cervical cancer with 266, 000 deaths annually [1]. Until now, radical hysterectomy with an open abdominal approach was the predominant modality for the treatment of early cervical cancer [2]. After 1992, with the development of laparoscopic approach, minimally invasive surgery (MIS, i.e., laparoscopy or robotic surgery) for radical hysterectomy to treat cervical cancer has been accepted widely as a standard treatment for early-stage cervical cancer [3].**

**Surprisingly, the results of Laparoscopic Approach of the Cervix (LACC) clinical trial showed that minimally invasive radical hysterectomy was associated with lower rates of disease-free survival and overall survival compared with open surgery in 2018 [4]. After that, the open abdominal approach was defined as the “standard and recommended approach to radical hysterectomy” for cervical cancer by the National Comprehensive Cancer Network (NCCN) guidelines [5]. Therefore, discussing the surgical complications have to be done clarifying better the actual role of MIS and laparotomy in cervical cancer.**

**Till date, the advantages of MIS over laparotomy for management of gynecological diseases have been cited in the literature to included less blood loss, shorter hospital stay, and faster recovery [6-8]. Similarly, most previous studies on this subject also showed that robotic surgery has the advantages of providing a three-dimensional perspective and more accurate surgical positioning than laparotomy [9-11]. However, MIS was also associated with its complexity of operation, longer learning curve, and higher cost than laparotomy. Therefore, there is no good evidence of the overall advantage of one surgical modality over another, and it is uncertain whether MIS is justifiable in terms of safety and efficacy, due to the small sample sizes, the low-quality of previous studies, and the limited number of randomized controlled trials (RCTs).**

**As for complications, many previous studies showed that MIS and open radical hysterectomy (ORH) have no difference in terms of intraoperative and postoperative complications [12]. With further development of instruments and skills, several studies found that MIS was associated with lower rate of** **intraoperative and postoperative complications than laparotomy [13]. Unfortunately, till date, it is unclear whether the rates of individual complications in MIS are also less than what are seen in laparotomy. Further emphasizing the severity of complications, which are a key factor in the evaluation of cervical cancer.**

**The aim of this meta-analysis was to compare the published rates of common intraoperative and postoperative complications between ORH and MIS in order to provide valid evidence for evaluating the advantages of different surgical procedures for managing cervical cancer.**

**Response to Reviewer #2**

**Q1:** The title of this manuscript does not well represent the topic.

Response: Thank you for these precious comments and suggestions. Thank you very much for your advice, we also found that the previous title cannot provide more details to indicate the aim of this meta-analysis. The title of this manuscript was changed to **“Comparison of the complications between minimally invasive surgery and open surgical treatments for early-stage cervical cancer: a systematic review and meta-analysis”**

**Q2:** Why did the authors choose to focus only on studies that compare the two (or three) surgical methods and exclude case series? Given that only one study was randomized, it seems that they would have had a much larger number of articles without losing substantial rigor.

Response: Thank you for these precious comments and suggestions. This was a good advice for us, we have discussed this point in discussion. In the future, we can further focus on all articles including case series

**There are limitations to this meta-analysis. First, included studies were primarily non-randomized studies, which could not provide high-quality evidence. Furthermore, our study did not include single-arm studies, which can lead to the bias of the result.**

**Q3:** It would be helpful to ground more general readers into the stage at which women would be candidates for surgical treatment, and how that may differ in LMICs compared to HICs where there are more resources available for radiation.

Response: Thank you for these precious comments and suggestions. The aim of this meta-analysis was to compare the difference of complications between MIS and open surgical treatments. We did not discuss this question which you bring out about the difference in LMICs compared to HICs where there are more resources available for radiation. Furthermore, we are so grateful for your suggestion, the difference of complications in LMIC for cervical cancer have been published in 2019, Refer to: PMID: 31269024. But the difference of complications between LMICs and HICs were not reported. This may be a good idea worthy of further study.

**Q4:** Line 87: The aim of the meta-analysis was to compare the published rates of common intra-operative and post-operative complications.
Response: Thank you for these precious comments and suggestions. We have revised this expression.

**Q5:** Was there a reason that the authors did not abstract the disease stage? Was this not presented in the published studies?

Response: Thank you for your comments. We have extracted the details of the disease stage into the form about characteristics of studies.

**Q6:** What does BMI mean in this context? Do you mean that in some studies, median BMI was reported vs mean vs no BMI description?

Response: Thank you for your comments. It was my mistake that I did not make this sentence clear. However, considering the statistic of BMI was not used for analysis, we choose to **delete line169-171**.

**Q7:** Lines 213-217: This needs to be worded more clearly, as the authors are stating that there is an increased odds of aggregate fistula formation, but when disaggregated, the odds of formation of individual fistula types were not increased, likely due to the small numbers in each category. The word “overall” in line 216 is confusing.

Response: Thank you for your comments. I have revised this part as **“In a stratified analysis (S2. Appendix), in an attempt to further determine the difference in fistula complications, we also analyzed complication with different types of fistula, including vesicovaginal, rectovaginal, ureterovaginal and urinary fistula, with ORs of 1.55 (95%CI=0.59-4.06, P=0.376), 2.88 (95%CI=0.44-18.70, P=0.269), 1.60 (95%CI=0.59-4.34, P=0.353), and 1.25 (95%CI=0.53-2.97, P=0.612) respectively. Interestingly, there was no significant difference in risk of the individual fistula types between MIS and ORH.”**

**Q8:** Discussion: The second sentence in the discussion states that the authors aimed to offer new opinions for prognostic indicators in MIS—this was not described in the methods and none of the results would suggest prognostic indicators were evaluated.

Response: Thank you for your comments. According to your advice, I have revised this part as **“We aimed to provide a basis for the selection of optimal surgical methods, as well as offer new opinions for actual role of MIS in cervical cancer.”**

**Q9:** Discussion: Line 281: would change that sentence to read that intestinal obstruction is often related to abdominal adhesions, which are more likely to form after abdominal surgery. I would avoid the phrase “indirectly proved.”

Response: Thank you for your comments. I revised this sentence as **“Intestinal obstruction is often related to abdominal adhesions, which are more likely to form after abdominal surgery”**

**Q10:** Discussion: Lines 297-302: There was no difference in fistula rates between ORH and MIS. Given the low numbers of fistulas seen overall, I would avoid making the over-conclusions that the authors may be doing in lines 298-302.

Response: Thank you for your comments. We have revised this part.

**Q11:** Discussion: I would suggest that the entire paragraph 284-302 can be combined with the preceding two paragraphs, preferably the urinary complication paragraph. The second sentence, describing the incidence of complications as depending on the surgeon’s skills, is general to all of the complications, not just fistula formation.

Response: Thank you for your comments. We have revised this part as **“Taken together, the surgeon proficiency may be a factor in determining the rates of complications. Regrettably, this meta-analysis was not able to provide a comparison between surgeons. Furthermore, the learning curve could play an important role in complications between different surgical modalities, and MIS was associated with a longer learning curve than ORH because of the complexity of surgical procedure, and also might have influenced complication rates. [62,63] The use of surgical instruments was related to viscus injuries, which may be caused by thermal injury, due to the high temperature of the surgical instruments resulting in the damage of submucosal or deeper tissues of the bladder, intestines, and bowel. Previous studies have evaluated the thermal injury of bowel in laparoscopic approach. [62] It must be taken that thermal injury is an inherent risk of the technique during radical hysterectomy, and therefore surgeon should pay attention to this issue. Overall, these factors were associated with the incidence of intraoperative and postoperative complications.”**

**Q12:** Discussion: Additional limitations that were not described include the lack of data on the surgeon level or years of experience, and additional patient morbidities (in addition to prior surgeries).

Response: Thank you for your comments. We discussed this point in the discussion.

**Taken together, the surgeon proficiency may be a factor in determining the rates of complications. Regrettably, this meta-analysis was not able to provide a comparison between surgeons.**

**Q13:** Table 2 needs a more descriptive title.

Response: Thank you for your comments. We revised this title as “**The subgroup analysis of laparoscopic types between MIS and ORH in intraoperative and postoperative overall complications”**

**Q14:** Figure 1: Include the reasons that articles were excluded and the #s per reason at the abstract and full-text stage in the flowchart.

Response: Thank you for your comments. We revised this flowchart.
